# Comparison of oxygen-15 PET and transcranial Doppler CO_2_-reactivity measurements in identifying haemodynamic compromise in patients with symptomatic occlusion of the internal carotid artery

**DOI:** 10.1186/2191-219X-2-30

**Published:** 2012-06-09

**Authors:** Suzanne Persoon, L Jaap Kappelle, Bart N M van Berckel, Ronald Boellaard, Cyrille H Ferrier, Adriaan A Lammertsma, Catharina J M Klijn

**Affiliations:** 1 Department of Neurology, Rudolf Magnus Institute of Neuroscience, University Medical Center Utrecht, G03.232, Utrecht, Heidelberglaan 100, 3584 CX, The Netherlands; 2 Department of Nuclear Medicine & PET research, VU University Medical Center, Amsterdam, De Boelelaan 1117, 1081 HV, The Netherlands; 3 Department of Clinical Neurophysiology, University Medical Center Utrecht, Utrecht, Heidelberglaan 100, 3584 CX, The Netherlands

**Keywords:** carotid artery disease, haemodynamic, PET, transcranial Doppler, stroke

## Abstract

**Background:**

Transcranial Doppler (TCD) CO_2_-reactivity and oxygen-15 positron emission tomography (PET) have both been used to measure the cerebral haemodynamic state in patients who may have a compromised blood flow. Our purpose was to investigate whether PET and TCD identify the same patients with an impaired flow state of the brain in patients with internal carotid artery (ICA) occlusion.

**Methods:**

Patients with recent transient ischaemic attack or minor ischaemic stroke associated with ICA occlusion underwent TCD with measurement of CO_2_-reactivity and oxygen-15 PET within a median time interval of 6 days.

**Results:**

We included 24 patients (mean age 64 ± 10 years). Seventeen (71%) patients had impaired CO_2_-reactivity (≤20%), of whom six had absent reactivity (0%) or steal (<0%) in the hemisphere ipsilateral to the ICA occlusion. PET of the perfusion state of the hemisphere ipsilateral to the ICA occlusion demonstrated stage 1 haemodynamic compromise (decreased cerebral blood flow (CBF) or increased cerebral blood volume (CBV) without increased oxygen extraction fraction (OEF)) in 13 patients and stage 2 (increased OEF) in 2 patients. In 12 patients (50%), there was agreement between TCD and PET, indicating haemodynamic compromise in 10 and a normal flow state of the brain in 2 patients. There was no significant correlation between CO_2_-reactivity and CBF ipsilateral/contralateral hemispheric ratio (*r* = 0.168, *p* value = 0.432), OEF ratio (*r* = −0.242, *p* value = 0.255), or CBV/CBF ratio (*r* = −0.368, *p* value = 0.077).

**Conclusions:**

In patients with symptomatic ICA occlusion, identification of an impaired flow state of the brain by PET and TCD CO_2_-reactivity shows concordance in only half of the patients.

## Background

The risk of recurrent ischaemic stroke in patients who present with transient ischaemic attack (TIA) or ischaemic stroke, associated with an internal carotid artery (ICA) occlusion, may be as high as 12% per year in case of a demonstrated compromised flow to the brain [1-3]. Because of this increased risk, revascularization surgery has been considered in these patients. ‘The Carotid Occlusion Surgery Study’ used PET to select patients with symptomatic ICA occlusion and high oxygen extraction fraction (OEF) for inclusion in their study that aimed to investigate whether extracranial-intracranial (EC/IC) bypass surgery prevents recurrent stroke [4]. The results showed that the 2-year risk of ipsilateral stroke did not differ between the surgical and non-surgical group (*p* = 0.78) despite an improvement in OEF ratio and a bypass patency of 96% at the last follow up [5]. Other types of revascularization, such as carotid endarterectomy of the contralateral ICA or of the ipsilateral external carotid artery, or surgery or stenting of the vertebral artery, for patients with symptomatic ICA occlusion may be considered as well, although firm evidence that these treatments reduce the risk of stroke is lacking [1,6-8].

The haemodynamic state of the brain can be subdivided into stage 0, the normal flow state of the brain; stage 1, the phase of vasodilatation to compensate for a decrease in blood flow towards the brain through cerebral autoregulation; and stage 2, the phase of autoregulation failure, with a compensatory rise in OEF [9]. Haemodynamic compromise stage 2 can only be demonstrated by positron emission tomography (PET) using oxygen-15-labelled tracers [10]. Previous oxygen-15 PET studies of patients with symptomatic ICA occlusion have shown a 2-year risk between 25% and 75% of recurrent ischaemic stroke in those with an increased OEF in comparison with a risk between 5% and 10% in patients without an increased OEF [2,3]. A disadvantage of PET scans with O-15 tracers is that this technique is not widely available and has a failure rate between 20% and 40% for obtaining complete quantitative data, mostly due to technical difficulties [2,9,11,12]. A widely available and cheap alternative for identification of patients with haemodynamic compromise is transcranial Doppler (TCD) with measurement of cerebrovascular reactivity [13,14]. Although cerebrovascular reactivity cannot identify patients with haemodynamic stage 2, it allows distinction between normal and a compromised haemodynamic state. Little is known about the agreement between oxygen-15 PET and TCD CO_2_-reactivity. If these two methods would identify the same patients as being at risk of future stroke, clinical trials may not need to be restricted to centres with PET facilities. The purpose of this study was to investigate whether, in patients with recent TIA or stroke associated with ICA occlusion, oxygen-15 PET parameters and TCD CO_2_-reactivity identify the same patients as having an impaired flow state of the brain.

## Methods

### Patients

We prospectively included 24 patients referred to the Department of Neurology, University Medical Center Utrecht, the Netherlands, between December 2004 and September 2009. Patients were included if they had transient or, at most, moderately disabling (modified Rankin scale ≤ 3) [15] neurological deficits associated with an ICA occlusion in the previous 3 months and complete oxygen-15 H_2_O, O_2_ and CO PET and TCD CO_2_-reactivity studies. Contrast angiography was performed to confirm occlusion of the ICA and to study collateral pathways [16]. Patients were excluded if there was evidence of arterial dissection or radiation vasculopathy as cause of the occlusion of the ICA. Magnetic resonance imaging of the brain was performed to investigate the presence of ischaemic lesions and included a three dimensional (3D) T1 image needed for PET image analysis. Six patients were excluded because of incomplete PET data due to either failure to insert the arterial cannula (*n* = 3) or technical difficulties (*n* = 3). Two other patients were excluded because TCD measurement failed due to an absent temporal bone window.

The institutional medical review board of the University Medical Center Utrecht approved the study protocol. All patients provided written informed consent.

### TCD CO_2_-reactivity

CO_2_-reactivity was measured by TCD using a Multi-Dop X device (DWL, Sipplingen, Germany) with two 2-MHz pulsed Doppler probes for insonation of cerebral vessels and a 4-MHz probe for the ophthalmic artery, as described previously [17]. After a standard TCD to locate the cerebral vessels, CO_2_-reactivity was measured simultaneously in both middle cerebral arteries (MCAs). Hypercapnia was induced by inhalation of a gas mixture containing 5% CO_2_ and 95% O_2_ (carbogene) through a mouthpiece connected to a respiratory balloon. A nose-clip ensured proper inhalation of carbogene. A spectral TCD recording of 5-s duration was acquired after breathing room air for 1 min and inhaling carbogene for 1.5 min. Readings of end-tidal CO_2_ and blood pressure were taken just before carbogene inhalation and after 1.5 min. The average change in end-tidal pCO_2_ was 12 mmHg (standard deviation (SD), 6 mmHg). CO_2_-reactivity after carbogene inhalation was calculated as the relative (percentage) change in blood flow velocity (BFV) in the MCA from the mean baseline BFV, expressed as a percentage. CO_2_-reactivity ≤ 20% was considered as decreased as 20% corresponds with the mean CO_2_-reactivity minus 2 standard deviations in normal controls [18]. A CO_2_-reactivity of 0% was defined as absent reactivity and <0% as steal of blood flow from the hemisphere ipsilateral to the ICA occlusion by other areas.

### Positron emission tomography imaging

PET scans were acquired using an ECAT EXACT HR + scanner (CTI/Siemens, Knoxville, Tennessee) [19]. Each PET study consisted of three parts: (1) a dynamic emission scan (25 frames over 600 s) after intravenous administration of a bolus of 1,100 MBq ^15^O]H_2_O to measure cerebral blood flow (CBF), (2) a dynamic emission scan (20 frames over 600 s) after a 30-s net inhalation of approximately 300 MBq ^15^O]O_2_ gas through a nasal cannula to derive oxygen consumption and calculate OEF, and (3) an emission scan (3 frames over 360 s) following a net inhalation of approximately 200 MBq ^15^O]CO gas to measure cerebral blood volume (CBV). All emission scans were collected in 3D acquisition mode. To allow for radioactive decay, an additional 5-min period between scans was included so that each administration was 15 min after the previous one. Finally, a 10-min transmission scan was acquired for attenuation and scatter correction purposes of the emission scans. All scans were reconstructed using a standard FORE + 2D filtered backprojection algorithm with a Hanning filter at Nyquist frequency. The arterial input function was measured continuously using an online blood sampling device [20]. In addition, at set times, manual samples were taken for calibration purposes and for assessment of plasma to whole blood ratios. Finally, the average arterial oxygen content was derived from blood gas analysis of three arterial samples. Further details of the scanning procedure can be found elsewhere [12]. Normal CBF, OEF, and CBV values were derived from 14 scans in seven healthy subjects (mean age 66 ± 7 years; five men) who underwent a PET scan on two separate occasions with a median time interval of 7 days, as published previously [12]. Mean values of both scans for each healthy subject were used, and the standard deviation was calculated. CBF, OEF, and CBV values in patients were considered to be abnormal if they were beyond mean values of normal controls ±1.96 times the SD.

### Image analysis

Individual anatomical 3D T1 MR images were co-registered using summed ^15^O]H_2_O images. A standard template of flow territories of middle cerebral artery, anterior cerebral artery, and posterior cerebral artery [21] was warped onto the co-registered MR image using Automated Image Registration software [22], applying a non-rigid 12-parameter perspective warping. Statistical Parametric Mapping (SPM02, London, UK, application in Matlab 7.0.4; MathWorks, Inc., Natick, MD, USA) was used for segmentation of grey and white matter. Areas of infarction were excluded manually. Parametric CBF, OEF and CBV images were generated using in-house developed software (written in IDL, 6.2, ITT, Boulder CO, USA) [23].

### Data analysis

Patients were divided into haemodynamic stages based on their values in the grey matter of the MCA territory: patients with normal CBF (≥31.1 mL/min/100 mL), normal CBV (≤3.9 mL/100 mL), and normal OEF (≤55.7%) were classified as haemodynamic stage 0; patients with either decreased CBF or increased CBV (both signs of autoregulation), but normal OEF, as haemodynamic stage 1; and patients with increased OEF (>55.7%) as haemodynamic stage 2. In addition, CBV/CBF was calculated as a measurement of mean transit time. Absolute CBF, OEF, and CBV/CBF values in the MCA territory, together with their hemispheric ratios (ipsilateral/contralateral), were compared in patients with and without absent CO_2_-reactivity or steal (CO_2_-reactivity ≤ 0%) using the Student’s *t* test or, in case of non-parametric variables, the Mann–Whitney *U* test. The same analysis was performed in patients with and without decreased CO_2_-reactivity (CO_2_-reactivity ≤ 20%). In addition, Pearson or, in case of non-parametric variables, Spearman correlation coefficients were calculated to assess relationships of CBF, OEF, and CBV/CBF with CO_2_-reactivity.

## Results

**Figure 1:**
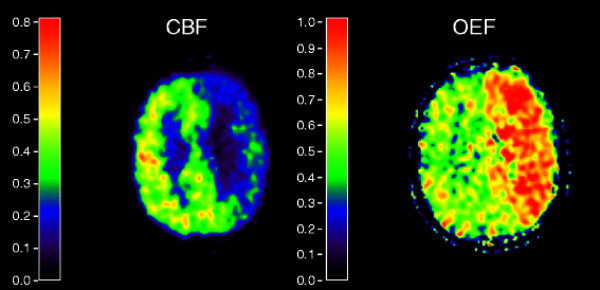
**Parametric images of cerebral blood flow and oxygen extraction fraction.** Parametric images of cerebral blood flow (CBF) and oxygen extraction fraction (OEF) measured using PET in a 69-year-old man who presented with a minor ischaemic stroke in the left hemisphere with an occlusion of the left internal carotid artery. PET images show decreased CBF and increased OEF in the left hemisphere, indicating haemodynamic failure (stage 2). Using transcranial Doppler, CO_2_-reactivity of the left middle cerebral artery was measured as −12%.

Clinical characteristics and angiogram findings of the 24 patients are shown in Table 1. The mean time between last symptoms and TCD was 33 ± 25 days. The median time between TCD and PET was 6 days (range 1 to 39). Seventeen patients had TCD before PET, and seven patients had TCD 7 days at the most after PET. Seventeen patients (71%) had impaired TCD CO_2_-reactivity, of whom three had absent CO_2_-reactivity and three showed a steal phenomenon. PET studies indicated that haemodynamic compromise was present in 15 patients (63%), of whom 13 patients were classified as haemodynamic stage 1 (with decreased CBF in 10, increased CBV in 3, and both decreased CBF and increased CBV in 2 patients) and 2 patients were classified as stage 2. As an example, Figure 1 shows parametric images of a patient with stage 2 haemodynamic failure. In 12 of the 24 patients (50%), TCD and PET showed agreement in haemodynamic status. In 10 of these 12 patients, haemodynamic compromise was demonstrated by both TCD and PET. In two patients, the flow state of the brain was normal in both TCD and PET. Of the 12 patients with different haemodynamic assessment in TCD and PET, 7 patients with impaired CO_2_-reactivity (−4%, 0%, 0%, 5%, 11%, 15%, and 19%, respectively) had a normal haemodynamic state (stage 0) in PET. Of five patients with normal CO_2_-reactivity, 4 were classified as stage 1 and one as stage 2 based on PET findings. The other patient with stage 2 haemodynamic failure based on PET showed steal on CO_2_-reactivity (−12%). In patients with agreement between PET and TCD, the median change in systolic blood pressure during TCD was 0 mmHg (interquartile range (IQR) 25), which did not differ from the median change in systolic blood pressure of 5 mmHg (IQR 10) in those without agreement (*p* = 0.850). The median change in diastolic blood pressure in patients with agreement between PET and TCD (0 mmHg, IQR 5) did not differ from the median change in those without agreement (3.5 mmHg, IQR 10; *p* = 0.257).

**Table 1:** **Clinical characteristics of patients with a symptomatic ICA occlusion (*n*** **= 24)**

**Characteristics**	**Number of patients (%)**	
Age (years, mean ± SD)	64	±10
Male	20	(83)
**Clinical features at presentation:**		
cerebral TIA	17	(71)
ischaemic stroke	7	(29)
Repeated symptoms after documented occlusion	22	(92)
Systolic blood pressure (mmHg, mean ± SD)	158	±28
Diastolic blood pressure (mmHg, mean ± SD)	86	±14
**Vascular risk factors:**		
Hypertension^a^	20	(83)
Hyperlipidaemia^b^	22	(92)
Diabetes mellitus	6	(25)
Cigarette smoking (current or in last 5 years)	13	(54)
History of stroke > 3 months ago	7	(29)
History of ischaemic heart disease	8	(33)
**MRI:**		
Ischaemic lesions in MCA territory ipsilateral to ICA occlusion:	11	(45)
endzone branche	3	
large subcortical (> 1.5 mm)	3	
cortical borderzone	5	
**Angiogram:**		
Bilateral ICA occlusion	1	(4)
Contralateral ICA stenosis ≥50%	9	(38)
Ipsilateral ECA stenosis ≥50%	6	(25)
Vertebral artery stenosis ≥50%	9	(38)
Collateral flow via anterior communicating artery	22	(92)
Collateral flow via ophthalmic artery	11	(46)
Collateral flow via posterior communicating artery^c^	20	(83)
Leptomeningeal collaterals^d^	17	(71)

^a^Hypertension was defined as blood pressure > 160/95 mmHg or current use of antihypertensive medication. ^b^Hyperlipidaemia was defined as either a history of hyperlipidaemia, current use on statins or levels of total cholesterol, triglycerids or high density lipoprotein cholesterol outside the normal ranges. ^c^Presence of collateral flow via the posterior communicating artery could not be judged in 1 patient. ^d^Presence of leptomeningeal collaterals could not be judged in 1 patient.

In Table 2, PET findings are compared between patients with and without impaired CO_2_-reactivity and between patients with and without absent reactivity or steal as measured by TCD. No significant differences were found in absolute CBF, OEF, and CBV/CBF values between patients with impaired and normal CO_2_-reactivity and between patients with and without absent CO_2_-reactivity or steal. A prolonged mean transit time in the symptomatic hemisphere in relation to the asymptomatic hemisphere (CBV/CBF hemispheric ratio) was found to be associated with an impaired CVR (*p* = 0.035).

**Table 2:** **Comparison of absolute ipsilateral PET values and hemispheric ratios with TCD CO**_**2**_**-reactivity**

	**All patients**	**Impaired CVR (≤20%)**	**Normal CVR (>20%)**	***p*value**	**Absent CVR or steal (≤0%)**	**No absent CVR or steal (>0%)**	***p*value**	**Test**
	***n*** **= 24**	***n*** **= 17**	***n*** **= 7**		***n*** **= 6**	***n*** **= 18**		
CO_2_-reactivity (%), mean ± SD	15.5 ± 15.9	7.0 ± 8.7	36.0 ± 9.0		−2.7 ± 4.8	21.6 ± 13.5		
CBF (mL/min/100 mL), mean ± SD	31.9 ± 5.6	32.3 ± 6.0	30.9 ± 4.8	0.572	30.8 ± 5.0	32.3 ± 5.9	0.598	*t*
CBF ratio, mean ± SD	0.88 ± 0.09	0.87 ± 0.08	0.90 ± 0.12	0.461	0.84 ± 0.09	0.89 ± 0.09	0.178	*t*
OEF (%), median (IQR)	45.1 (42.2 to 50.9)	45.0 (42.5 to 50.1)	47.8 (40.2 to 55.2)	0.634	47.6 (42.1 to 58.4)	45.0 (41.5 to 50.8)	0.424	*U*
OEF ratio, median (IQR)	1.06 (1.00 to 1.11)	1.06 (1.01 to 1.11)	1.08 (0.97 to 1.12)	0.949	1.09 (1.04 to 1.26)	1.05 (0.99 to 1.10)	0.116	*U*
CBV/CBF, mean ± SD	0.10 ± 0.03	0.10 ± 0.03	0.11 ± 0.03	0.563	0.09 ± 0.04	0.10 ± 0.02	0.266	*t*
CBV/CBF ratio, mean ± SD	1.27 ± 0.18	1.32 ± 0.15	1.15 ± 0.21	0.035	1.36 ± 0.15	1.24 ± 0.18	0.174	*t*

CBF, cerebral blood flow; CBV, cerebral blood volume; CVR, cerebrovascular reactivity; IQR, interquartile range; OEF, oxygen extraction fraction; *t*, Student’s *t* test; *U*, Mann–Whitney test.

**Figure 2:**
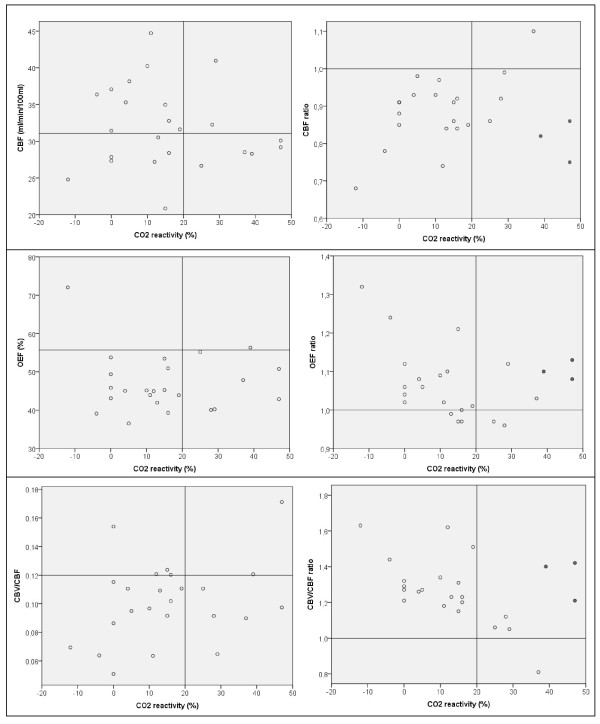
**Relationship of CO**_**2**_**-reactivity with PET values.** Scatter plots showing the relationships of CO_2_-reactivity with CBF (upper panel), OEF (middle panel), and CBV/CBF (lower panel). Within each panel, absolute values are shown on the left and ipsilateral/contralateral ratios on the right. The grey dots in the panels on the right indicate the three outliers.

Figure 2 illustrates that, on visual inspection, ipsilateral/contralateral ratios of CBF, OEF, and CBV/CBF corresponded better with CO_2_-reactivity than absolute ipsilateral values of CBF, OEF, and CBV/CBF. Nevertheless, there was no significant correlation between CO_2_-reactivity and CBF hemispheric ratio (Pearson *r* = 0.168, *p* = 0.432), OEF hemispheric ratio (Spearman *r* = −0.242, *p* = 0.255), and CBV/CBF hemispheric ratio (Pearson *r* = −0.368, *p* = 0.077). Remarkably, three patients with high CO_2_-reactivity (indicated by grey dots in Figure 2) were the main reason for the poor correlations. Two of them were males aged 45 and 73 years, and the female patient was 76 years old. The number of days between TCD and PET was at most 7 days in these three patients. Only in one of the three patients, blood pressure during TCD increased with 50 mmHg systolic and 20 mmHg diastolic; in the other two, the systolic blood pressure remained stable or decreased with 15 mmHg, and the diastolic blood pressure remained stable. After excluding these three patients in a *post hoc* analysis, significant correlations were obtained between CO_2_-reactivity and CBF ratio (Pearson *r* = 0.563, *p* = 0.008), OEF ratio (Spearman *r* = −0.542, *p* = 0.011), and CBV/CBF ratio (Pearson *r* = −0.677, *p* = 0.001).

## Discussion

The main finding of this study is that, in patients with symptomatic ICA occlusion, identification of the presence or absence of haemodynamic compromise by oxygen-15 PET and TCD CO_2_-reactivity corresponds in only half of the patients. This is important as the presence of haemodynamic compromise has been shown to predict recurrent stroke [1-3] and may play a role in the decision whether or not to advise revascularization surgery. We did not find a difference in the change in blood pressure during TCD between patients with and without agreement between PET and TCD. Therefore, it is unlikely that an increase in blood pressure during TCD resulting in an overestimated CO_2_-response is responsible for the incongruent findings between PET and TCD. After excluding three patients with high CO_2_-reactivity, a correlation with the various ipsilateral/contralateral PET ratios was found, albeit only at a moderate level. It is not clear why these three patients were outliers as age or time between TCD and PET were not different from the other patients.

Previous studies investigating the relationship between cerebrovascular reactivity and CBF or OEF measured by PET also included patients with other types of vascular obstruction other than ICA occlusion and reported conflicting results (Table 3). Only one previous study made the same comparison as we did between TCD CO_2_-reactivity with oxygen-15 PET, but they selected 22 patients with hypertension and diabetes. In concordance with our findings, this study did not find an association between increased OEF and decreased TCD CO_2_-reactivity [24]. Other studies have reported an association between impaired cerebrovascular reactivity to hypercapnia or acetazolamide measured with SPECT or stable-xenon CT studies and increased PET OEF [25-29].

**Table 3:** Overview of studies comparing cerebrovascular reactivity with CBF or OEF

**Author**	**Method 1**	**Method 2**	**Sample (*n*)**	**ICA occlusion**	**Symptoms**	**Agreement between methods**
Fujimoto et al. 2002 [29]	IMP-SPECT + ACZ	^15^O-PET	53	18	47	Haemodynamic compromise in 75% by method 1, 32% by method 2 (increased OEF, stage 2), and 64% by method 2 (stage 1).
						Correlation CVR (asymmetry index) with CBF/CBV *r* = 0.31 (*p* < 0.05)
						Correlation CVR with OEF *r* = −0.64 (*p* < 0.0001)
Herold et al. 1988 [25]	Xenon-SPECT + CO_2_	^15^O-PET	21	19	7	Haemodynamic compromise in 29% by method 1 and 19% by method 2 (increased OEF)
						Correlation CVR with CBF/CBV *r* = 0.575 (*p* < 0.01)
						Correlation CVR with oxygen extraction ratio *r* = −0.573 (*p* < 0.01)
Imaizumi et al. 2002 [28]	IMP-SPECT + ACZ	^15^O-PET	27	9	22	No data on the number of patients with haemodynamic compromise
						Correlation CVR with CBV *r* = −0.31 (*p* < 0.01)
						Correlation CVR with OEF *r* = −0.55 (*p* < 0.0001)
Nariai et al. 1995 [26]	Xenon CT + ACZ	^15^O-PET	11	4	10	No data on number of patients with haemodynamic compromise
						Correlation CVR with CBV *r* = −0.50 (*p* = 0.02)
						Correlation CVR with OEF *r* = −0.65 (*p* = 0.001)
Nemoto et al. 2004 [27]	Xenon CT + ACZ	^15^O-PET	12	12	12	Haemodynamic compromise in 50% by method 1 and 17% by method 2 (increased OEF)
						Correlation CVR with OEF *r* = −0.57 (*p* = 0.001)
Sugimori et al. 1995 [24]	TCD + CO_2_	^15^O-PET	22	2	7	Haemodynamic compromise in 52% by method 1 and 9% by method 2 (increased OEF)
						Correlation CVR with CBF *r* = 0.47 (*p* < 0.05)
						Correlation CVR with OEF *r* = 0.20 (ns)

TCD, transcranial Doppler; CO_2_, carbon dioxide; ACZ, acetazolamide; CT, computed tomography; PET, positron emission tomography; CVR, cerebrovascular reactivity; CBF, cerebral blood flow; OEF, oxygen extraction fraction; CBV, cerebral blood volume.

The results of the present study indicate that, in patients with recently symptomatic ICA occlusion, TCD measurements of the haemodynamic state of the brain cannot be replaced by oxygen-15 PET and vice versa for identification of patients with haemodynamic compromise. This may be explained by important differences between TCD and oxygen-15 PET. First, TCD measures blood flow velocity in the MCA itself, whereas PET measures perfusion at the level of the brain tissue. The method of TCD relies on the assumption that changes in flow velocity are directly proportional to changes in CBF. For that to be true, the cross-sectional area of the insonated artery needs to remain constant [30,31]. In contrast, PET directly measures CBF, CBV, and OEF, which are components of the autoregulation itself. In addition, TCD measures changes in flow velocity in response to hypercapnia, whereas PET, according to the scan protocol in this study, measures haemodynamic parameters at rest, i.e., without vasodilatory stimuli. Of the previous studies that compared the CBF response after a vasodilatory stimulus measured by SPECT or stable-xenon CT with TCD reactivity [32-35], one study of 38 patients with ICA stenosis or occlusion found only a weak relation [32], and others reported moderate to good correlations between CBF response after a challenge and TCD cerebrovascular reactivity [33-35]. It is possible that the agreement between TCD and PET in our study would have been better if a vasodilatory stimulus had also been included in the PET studies. An advantage of PET is that it does not only provide information on perfusion in the MCA territory but also on the flow state of the other vascular territories of the brain. TCD and PET do not only measure different physiologic parameters but also use quite different techniques. As a gold standard for measuring the flow state of the brain is not yet available, the sensitivity and specificity of TCD and PET cannot be determined [1,10].

A strength of the present study in comparison with previous reports is that a more homogenous population of patients was included as all had recent symptoms of the hemisphere ipsilateral to an ICA occlusion. In addition, 92% of patients had ischaemic symptoms after documented occlusion, which is an important clinical risk factor for recurrent ischaemic stroke [36]. Furthermore, in contrast to some previous studies [24,25], the majority of patients in our cohort showed haemodynamic compromise measured by TCD or PET. It is in this subcategory of patients that accurate identification of haemodynamic compromise has implications for prognosis and possible treatment decisions.

This study has some limitations. First, the patients were not investigated by TCD and ^15^O-PET on the same day. Compromised cerebral perfusion can improve over time [37]. Although the majority of patients (88%) had TCD and PET within 2 weeks, the time interval between TCD and PET may have contributed to the poor agreement.

Second, the classification in haemodynamic stages may be a matter of debate. In the model of Derdeyn et al. [9], stage 1 was defined as a slight decrease of CBF and slight increase of OEF with or without increase of CBV, but a cutoff value for a *slight* difference was not provided. In another clinical PET study, the patients were only divided into two groups with a normal or increased OEF [3]. We defined stage 1 as a decreased CBF or increased CBV as both are signs of autoregulation. This study shows that both the comparison of haemodynamic stages by PET and TCD, as well as absolute values obtained by PET and TCD, did not show agreement.

Third, ten patients had an additional stenosis or occlusion in the contralateral ICA, which may have influenced the results in hemispheric ratios. Fourth, this study has a relatively small sample size. However, previous PET studies in relation to cerebrovascular reactivity were even smaller consisting of at most 19 patients with ICA occlusion [25].

## Conclusions

The present study shows that, in patients with symptomatic ICA occlusion, identification of presence or absence of haemodynamic compromise by oxygen-15 PET and TCD CO_2_-reactivity corresponds in only half of patients. In future trials, PET and TCD CO_2_-reactivity measurements cannot be used according to local preference to identify patients with haemodynamic compromise for study inclusion.

## Competing interests

The authors declare that they have no competing interests.

## Authors’ contributions

SP participated in the design of the study, performed the statistical analysis, and drafted the manuscript. BNMB, RB, and AAL were involved in the acquisition and interpretation of the PET studies and critically revised the manuscript. CHF was involved in the acquisition and interpretation of the TCD studies and critically revised the manuscript. LJK and CJMK participated in the design of the study, interpreted the data and critically revised the manuscript. All authors read and approved the final manuscript.
